# Imputation and Missing Indicators for Handling Missing Longitudinal Data: Data Simulation Analysis Based on Electronic Health Record Data

**DOI:** 10.2196/64354

**Published:** 2025-03-13

**Authors:** Molly Ehrig, Garrett S Bullock, Xiaoyan Iris Leng, Nicholas M Pajewski, Jaime Lynn Speiser

**Affiliations:** 1 Department of Biostatistics and Data Science, Wake Forest University School of Medicine, Medical Center Blvd, Winston Salem, NC, 27157, United States, 1 3367133469

**Keywords:** missing indicator method, missing data, imputation, longitudinal data, electronic health record data, electronic health records, EHR, simulation study, clinical prediction model, prediction model, older adults, falls, logistic regression, prediction modeling

## Abstract

**Background:**

Missing data in electronic health records are highly prevalent and result in analytical concerns such as heterogeneous sources of bias and loss of statistical power. One simple analytic method for addressing missing or unknown covariate values is to treat missingness for a particular variable as a category onto itself, which we refer to as the missing indicator method. For cross-sectional analyses, recent work suggested that there was minimal benefit to the missing indicator method; however, it is unclear how this approach performs in the setting of longitudinal data, in which correlation among clustered repeated measures may be leveraged for potentially improved model performance.

**Objectives:**

This study aims to conduct a simulation study to evaluate whether the missing indicator method improved model performance and imputation accuracy for longitudinal data mimicking an application of developing a clinical prediction model for falls in older adults based on electronic health record data.

**Methods:**

We simulated a longitudinal binary outcome using mixed effects logistic regression that emulated a falls assessment at annual follow-up visits. Using multivariate imputation by chained equations, we simulated time-invariant predictors such as sex and medical history, as well as dynamic predictors such as physical function, BMI, and medication use. We induced missing data in predictors under scenarios that had both random (missing at random) and dependent missingness (missing not at random). We evaluated aggregate performance using the area under the receiver operating characteristic curve (AUROC) for models with and with no missing indicators as predictors, as well as complete case analysis, across simulation replicates. We evaluated imputation quality using normalized root-mean-square error for continuous variables and percent falsely classified for categorical variables.

**Results:**

Independent of the mechanism used to simulate missing data (missing at random or missing not at random), overall model performance via AUROC was similar regardless of whether missing indicators were included in the model. The root-mean-square error and percent falsely classified measures were similar for models including missing indicators versus those with no missing indicators. Model performance and imputation quality were similar regardless of whether the outcome was related to missingness. Imputation with or with no missing indicators had similar mean values of AUROC compared with complete case analysis, although complete case analysis had the largest range of values.

**Conclusions:**

The results of this study suggest that the inclusion of missing indicators in longitudinal data modeling neither improves nor worsens overall performance or imputation accuracy. Future research is needed to address whether the inclusion of missing indicators is useful in prediction modeling with longitudinal data in different settings, such as high dimensional data analysis.

## Introduction

Electronic health record (EHR) data have many analytic uses, including patient monitoring, clinical decision support, quality improvement projects, and research initiatives [1]. However, missing data are pervasive in EHRs because these systems were largely designed for the purposes of billing and because of the fragmented nature of health care in the United States where patients often use multiple health systems with disparate EHR systems. The incomplete nature of the EHR creates significant potential for bias for research studies leveraging real-world data [2]. Statistically, missing data may be considered ignorable when they are missing completely at random (MCAR) or missing at random (MAR). A recent study illustrated that more than 1 missing mechanism may be present for EHR data, and the assumption that all missing data are MAR is generally not plausible [3]. Recent work by Hu et al [4] indicated that clinical EHR data were consistent with a mixture of random and nonrandom mechanisms. For example, a white blood cell count test was less likely to be ordered for patients who were clinically doing well (eg, lack of collection).

Current approaches to handle missing data include complete case analysis, imputation, and nonimputation approaches such as the use of missing indicators. These approaches vary in terms of their appropriateness depending on untestable assumptions about the mechanisms generating missing values. A detailed discussion of statistical approaches to handling missing data can be found in the TRIPOD (Transparent Reporting of a Multivariable Prediction Model for Individual Prognosis or Diagnosis) checklist [5,6]. Complete case analysis is a common method in which observations with missing values in any of the analysis variables are listwise deleted. If data are MCAR, complete case analysis may be appropriate, but if data are MAR or missing not at random (MNAR), complete case analysis can result in biased estimates. Independent of the missingness mechanism, complete case analysis results in a loss of statistical power by reducing the number of available observations [7,8].

Imputation is another commonly used method for handling missing data and involves using observed data to estimate and fill in values that are missing, typically through regression approaches that model the variable with missingness as the outcome with the other variables in the dataset as predictors. While this method retains all observations in the dataset and reduces bias when data are MAR, regression imputation underestimates the SE of the model parameters and therefore overestimates precision [8,9]. Multiple imputation overcomes the limitations of regression imputation by generating multiple imputed values for each missing value. By separately analyzing each dataset and combining the outputs to obtain an overall point estimate and corresponding SE, variability estimates are more accurate and the analysis accounts for the uncertainty caused by missingness [9,10]. However, the appropriate imputation strategy may depend on both the type of missingness and the objective of the analysis. One recent study has shown that regression imputation performs as well as multiple imputation when the ultimate goal is prediction rather than statistical inference or model interpretation [11]. Another study found that for logistic regression, regression imputation was comparable with multiple imputation in terms of model performance with a low percentage of missingness [12]. However, none of the imputation methods are unbiased or recommended for nonignorable missing data.

A third approach is the missing indicator method, which adds a binary predictor to the model that takes the value of 1 if the value of a certain variable is missing and zero if the value is not missing, therefore, taking advantage of the information contained in missingness itself [13]. The use of missing indicators has been introduced as a method when missingness in informative, or when the presence or absence of missingness adds prognostic information to a model. Although this is a simple method for potentially leveraging information about missingness, it increases the number of predictor variables to be included, which may not be ideal for high-dimensional datasets, datasets with many predictors, or situations where significant model flexibility is desired (ie, semiparametric models that use basis functions or splines to flexibly model continuous predictors such as vital signs or laboratory values).

There is still a lack of consensus on the appropriateness of the missing indicator method for handling missing data for clinical prediction modeling [14]. One concern is the creation of a negative feedback loop between the model and the providers using the model for decision support. When an individual knows that taking or not taking a certain measurement is informative, their decision to take the measurement could hypothetically be impacted [13,15], or the model may simply reiterate a clinical suspicion or decision that has already occurred, such as a recent prediction model for the early detection of sepsis [16]. An example for this is the decision to order certain specialized laboratory tests. In addition, prediction models that use the missing indicator method must be consistently monitored and revised due to how quickly patient medical data and factors that affect physician decision-making change [15]. However, other work has found that the missing indicator method could improve predictive performance [14,17]. One study found that the addition of missing indicators, which signaled the presence or absence of a laboratory test result, to observed measurements improved area under the receiver operating characteristic curve (AUROC) when predicting clinical outcomes [17]. Missing indicators have been shown to increase predictive performance when missingness is informative, with the effectiveness of the method increasing as the informativeness of missingness increased [14]. The same study found that the missing indicator method did not harm predictive performance when missingness was uninformative. This is an important distinction, as it is not possible to empirically test whether missingness is informative [18].

There is currently a gap in knowledge regarding the effectiveness of including missing indicators in longitudinal data modeling, specifically whether missing indicators improve model performance and the quality of model-based imputations. The setting of longitudinal repeated measures and clustered data is an important context for the missing indicator method because the correlation within clusters may be leveraged to increase the imputation accuracy and model performance, particularly in the case of data that are MNAR. However, we are not aware of work that has investigated the missing indicator method in this setting.

We aimed to assess the missing indicator method for longitudinal, repeated-measures data using a simulation study mimicking real-world EHR data. In section 2, we detail the methods we used to generate the synthetic longitudinal data, including fixed and repeated measures of predictors for MAR and MNAR missing data patterns, and we define outcome metrics used to assess performance and imputation quality. In section 3, we present results aggregated across the simulation runs for models with and with no missing indicator variables. In section 4, we discuss the results and implications of the study, compare our study with prior studies, and consider the strengths and limitations of this work.

## Methods

### Study Design

This study is a simulation study in which missing indicator variables in imputation and modeling were evaluated under different missing data mechanisms (MAR and MNAR). We follow the simulation study guidelines suggested by Morris and colleagues [19]. Analyses were performed with R (version 4.2.1; The R Project for Statistical Computing). All code is available on our GitHub repository [20]. We use the following R packages in our analysis: *bindata* [21], *MASS* [22], *tidyverse* [23], *lme4* [24], *lmerTest* [25], *naniar* [26], *mice* [27], *broom.mixed* [28], *pROC* [29], *DescTools* [30], *missForest* [31], *table1* [32], *flextable* [32], *skimr* [32], *sjPlot* [33], *gridExtra* [34], *grid* [35], and *car* [36].

### Data-Generating Mechanisms

This study focuses on a mixed effects logistic regression model that uses a binary outcome simulated to represent whether or not patients experienced a fall since their last visit. A total of 250 patients were simulated, each with 5 visits. Medical history variables, demographic variables, fall-specific variables, and variables intended to add noise to the model were simulated. We simulated the data to represent EHR data that may be used to develop models for falls in older adults. Predictors of falls were simulated based on previous research [37] and represent a combination of fixed, patient-level variables and visit-level variables that are collected repeatedly. The fixed variables included sex and comorbidities (diabetes, dementia, hypertension, and urinary incontinence), all of which may be related to falls in older adults. The visit-level variables included BMI, gait speed, single-leg balance, and use of medications (pain or depression), again representing variables that could be associated will falls in older adults. Table 1 lists all variables in the dataset and describes how they were simulated. We include summaries of the variables for one of the simulated datasets in Multimedia Appendix 1. For more details, including parameter values, see the code on GitHub.

**Table 1.** Variable list and description.

Variables	Data generation and description
Patient-level variables	
Birth sex, diabetes, dementia, hypertension, and urinary incontinence	Binary random variables simulated with the bindata R package
Age	Continuous with mean dependent on number of chronic conditions (ie, number of the following conditions: diabetes, dementia, hypertension, and urinary incontinence)
Visit-level variables	
Visit	Discrete, 5 visits for each patient.
BMI	Continuous, simulated with a linear mixed effects model with age, diabetes, hypertension, and birth sex as predictors. Random intercept for patient ID and random error included.
Gait speed	Continuous, simulated with a linear mixed effects model with age, BMI, diabetes, and birth sex as predictors. Random intercept for patient ID and random error included.
Single-leg balance	Continuous, simulated with a linear mixed effects model with age, BMI, diabetes, dementia, and birth sex as predictors. Random intercept for patient ID and random error included.
Pain medication	Binary, probability simulated with expit function with age, sex, and diabetes as predictors in the model. A random intercept for patient was included in the model. The probability was then used to simulate a Bernoulli random variable where: 0=did not take pain medication since last visit 1=took pain medication since last visit
Depression medication	Binary, probability simulated with expit function with age, sex, and dementia as predictors in the model. A random intercept for patient ID was included in the model. The probability was then used to simulate a Bernoulli random variable where: 0 = did not take depression medication since last visit 1 = took depression medication since last visit
Junk 1‐5	Continuous random variables with means and SDs chosen at random.
Y	Binary outcome variable, probability simulated with expit function with all variables except the junk variables and visit as predictors. A random intercept for patient ID was also included in the model. The probability was simulated with and with no missing indicators included in the model. The probability was then used to simulate a Bernoulli random variable where: 0 = did not fall since last visit 1 = fall since last visit

The probability of the binary outcome was simulated in 2 different ways using the expit function. For both versions, the model included a random intercept for patient, and all variables except the junk variables, patient ID, and visit were included as predictors. The first version included missing indicator variables as predictors in the model, while the second did not. The outcome was a random Bernoulli variable with the probability of being one equal to the calculated probability for each visit. A total of 250 iterations were run, so 250 different datasets were created.

Missingness was induced for the visit-level continuous variables gait speed and single-leg balance, and for the binary variables pain medication and depression medication. Overall missing data percentages of 20% and 50% were simulated. Under the assumption of MAR, the probability that gait speed, single-leg balance, pain medication, and depression medication were missing for a specific visit was dependent on age, BMI, diabetes, and urinary incontinence. Specifically, the probability each variable was missing was simulated with the expit function where age, BMI, diabetes, and urinary incontinence were included as predictors. The intercept was changed to achieve different percentages of missing data. The probability was higher for older patients, patients with a larger BMI, and patients with diabetes or urinary incontinence. Missing indicators were created by defining Bernoulli random variables with the probability of being one equal to the probability of being missing, and indicator variables were created for each of the 4 variables. If the missing indicator was 1, the value of the corresponding variable was set to missing. Therefore, although all 4 variables had the same probability of being missing for each visit, different combinations of variables could be missing at each visit.

Under the MNAR missingness mechanism, the probability that gait speed, single-leg balance, pain medication, and depression medication were missing for a specific visit was dependent on the value of the variable itself. For gait speed and single-leg balance, if the value of the variable at a visit was less than the 25th percentile, the probability of the value being set to missing was .7 to target an overall missing percentage of 50% and .3 to target an overall missing percentage of 20%. Otherwise, the probability was zero. For pain medication and depression medication, if the value of the variable was 1 at a visit (indicating that the patient was taking the medication), the probability the value was set to missing was .4 to target an overall missing percentage of 50% and .1 to target an overall missing percentage of 20%. Otherwise, the probability was zero. Therefore, lower values of gait speed and single-leg balance were more likely to be missing. Similarly, if patients were taking pain medication or depression medication, these values were more likely to be missing. For all of the simulated scenarios, the outcome, all patient-level variables, and the remaining visit-level variable (BMI) were fully observed.

### Missing Data-Handling Strategies

Multivariate imputation via chained equations was performed using the *mice* package in R [27]. Regression imputation was performed using single imputation (ie, multiple imputation was not used because the purpose of the model is prediction). All variables in the dataset were included in the imputation model, including the outcome variable. The 2-level structure of the dataset was specified in the imputation model by denoting patient as the clustering variable. To impute gait speed and single-leg balance, a 2-level normal model was used. Values below zero were capped at zero. To impute pain and depression medication, a 2-level logistic model was used. When imputing a variable, the indicator for that variable was not included in the imputation model because in imputation only present data are used and the value of the indicator is 1 for all present data. The indicators for the other variables were included in the imputation model.

The outcome was calculated with a mixed effects logistic regression for both analyses that included and did not include missing indicator variables. All variables except visit were included in the model as predictors, and a random intercept for patient was also included in the model. Missing indicators were included in the model when they had also been included in the imputation model. The junk variables were included with the expectation that they would not be significant in the model. Complete case analysis was performed by deleting all observations with missing values prior to running the model. A summary of the different scenarios and models run is shown in Table 2.

**Table 2.** Summary of Modeling.

Outcome simulation and missing data mechanism		Target missing percentage	Imputation and modeling strategy
Missing indicators included in model for outcome simulation			
	MAR^a^	20	Missing indicators included in imputation and modeling No missing indicators included in imputation and modeling Complete case analysis
	MAR	50	Missing indicators included in imputation and modeling No missing indicators included in imputation and modeling Complete case analysis
	MNAR^b^	20	Missing indicators included in imputation and modeling No missing indicators included in imputation and modeling Complete case analysis
	MNAR	50	Missing indicators included in imputation and modeling No missing indicators included in imputation and modeling Complete case analysis
No missing indicators included in model for outcome simulation			
	MAR	20	Missing indicators included in imputation and modeling No missing indicators included in imputation and modeling Complete case analysis
	MAR	50	Missing indicators included in imputation and modeling No missing indicators included in imputation and modeling Complete case analysis
	MNAR	20	Missing indicators included in imputation and modeling No missing indicators included in imputation and modeling Complete case analysis
	MNAR	50	Missing indicators included in imputation and modeling No missing indicators included in imputation and modeling Complete case analysis

a MAR: missing at random.

b MNAR: missing not at random.

### Performance Metrics

We assessed models in terms of performance and imputation quality. To assess model performance, AUROC was calculated. To assess imputation quality for binary variables, the proportion of falsely classified imputations (PFC) was calculated [38], defined as the number of incorrect binary imputed values divided by the total number of imputed values. Lower proportions indicate better quality of imputations. To assess imputation quality for continuous variables, the normalized root-mean-square error (NRMSE) between the imputed values and the observed values was calculated. The root-mean-square error is normalized by dividing by the SD of the observed values (from the study by Stekhoven and Bühlmann [38]). Lower NRSME indicates better imputation quality. For each iteration and scenario, the PFC, NRMSE, and AUROC were stored and the average values were calculated across the simulation runs.

### Simulation Study Analysis Pipeline

**Figure 1.**
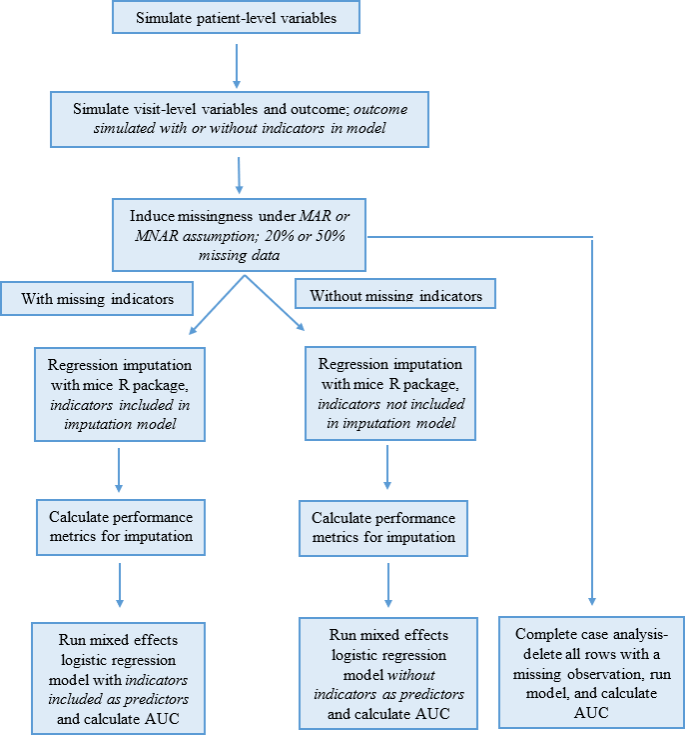
Data pipeline flowchart. AUC: area under the receiver operating characteristic curve; MAR: missing at random; MNAR: missing not at random.

Figure 1 provides an overview of the simulation and analysis performed in this study. The first key step is data generation, with patient-level variables generated first, then visit-level variables, and finally the outcome under the 2 underlined scenarios. Missingness is then induced under different mechanisms and at different percentages, and imputation occurs with and without the missing indicators in the imputation model. After the calculation of evaluation metrics, models were run with and without the missing indicators as predictors in the model—along with complete-case analysis—and the AUROC of each model was extracted. For models using imputation, NRMSE was calculated for continuous variables and PFC was calculated for binary variables. Results for each run of the simulation were aggregated, and averages of the performance metrics are presented.

## Results

Table 3 shows the average overall percentage of missing data and the SD for each scenario under the different missing mechanisms and data-generating mechanisms. For all scenarios, the actual missing percentage of data was slightly higher than the targeted amount.

**Table 3.** Missingness Percentages.

Outcome simulation and missing data mechanism		Target missing percentage	Actual missing percentage, mean (SD)
Indicators included in model for outcome simulation			
	MAR^a^	20	22.53 (1.09)
	MAR	50	52.18 (1.33)
	MNAR^b^	20	22.20 (1.08)
	MNAR	50	54.28 (1.33)
Indicators not included in model for outcome simulation			
	MAR	20	22.50 (1.06)
	MAR	50	52.34 (1.27)
	MNAR	20	22.35 (1.07)
	MNAR	50	54.29 (1.33)

a MAR: missing at random.

b MNAR: missing not at random.

### Imputation Quality

**Figure 2.**
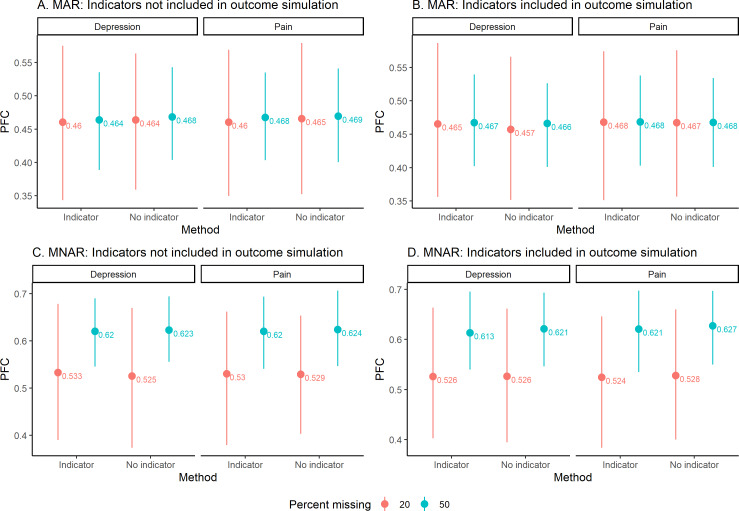
Average proportion of falsely classified imputations (PFC) for binary variables across iterations. The average value is indicated with a point, and the lines go to the 2.5th percentile and 97.5th quantiles. Panels A and B are for MAR data, when indicators are not included in the outcome simulation and when indicators are included in the outcome simulation. Panels C and D are for MNAR data, when indicators are not included in the outcome simulation and when indicators are included in the outcome simulation. MAR: missing at random; MNAR: missing not at random.

First, we present results related to imputation quality. We begin by assessing the PFC for the binary variables (Figure 2), in which higher PFC indicates a higher misclassification rate and therefore worse imputation quality. For MAR scenarios (Figure 2A and B), the PFC was about 46%‐47% for both pain and depression medication, regardless of whether indicators were used to simulate the outcome. There was little difference between the PFC at 20% of missing data compared with 50% of missing data. For MNAR scenarios (Figure 2C and D), the PFC was about 61%‐63% for both pain and depression medication at 50% of missing data and about 52%‐53% for 20% of missing data. PFCs comparing including missing indicators versus not including missing indicators were similar. The PFCs were higher for MNAR data than for MAR data.

**Figure 3.**
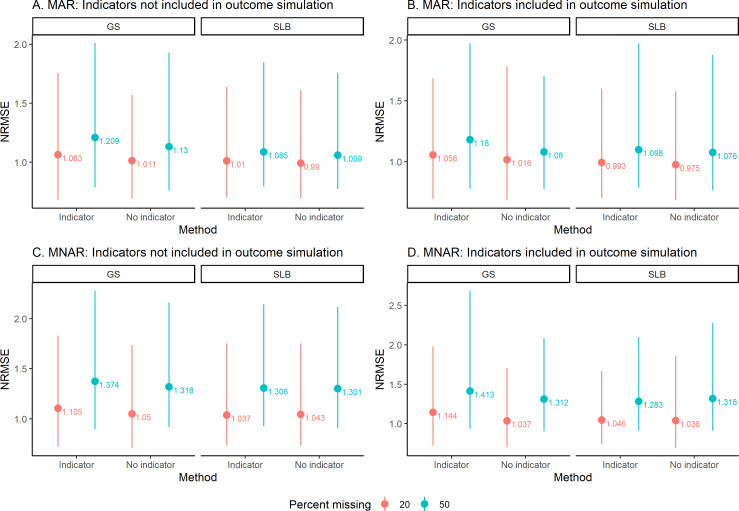
Average normalized root-mean-square error (NRMSE) for continuous variables across iterations. The average value is indicated with a point, and the lines go to the 2.5th percentile and 97.5th quantiles. Panels A and B are for MAR data, when indicators are not included in the outcome simulation and when indicators are included in the outcome simulation. Panels C and D are for MNAR data, when indicators are not included in the outcome simulation and when indicators are included in the outcome simulation. GS: gait speed; MAR: missing at random; MNAR: missing not at random.

Next, we assess NRMSE for continuous variables (Figure 3A-D), in which higher NRMSE indicates worse imputation quality. In general, the NRMSE of single-leg balance was lower than that of gait speed. For the variables gait speed and single-leg balance, NRMSE was higher when there was 50% of missing data compared with 20% of missing. NRMSE was higher in MNAR scenarios compared with MAR scenarios. Whether or not indicators were included when simulating the outcome resulted in similar NRMSE for the variables. The NRMSE for the imputation of gait speed was slightly larger when indicators were included for all scenarios, but for single-leg balance there was no clear pattern.

### Performance Evaluation

**Figure 4.**
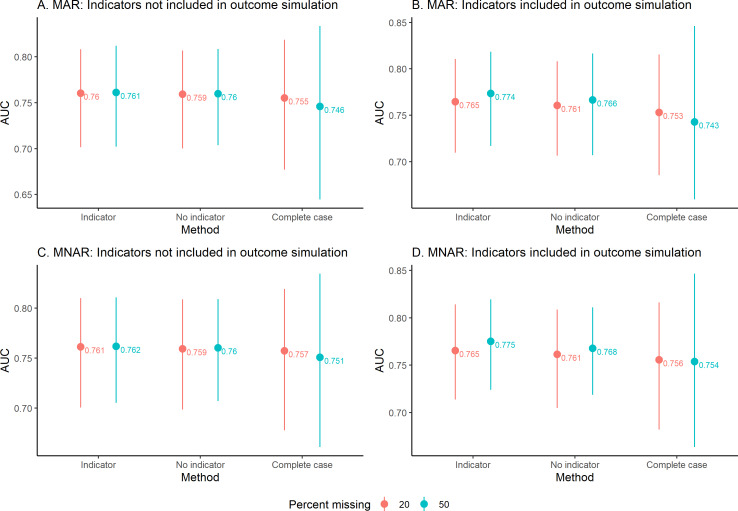
Average AUC comparison across iterations. The average value is indicated with a point, and the lines go to the 2.5th percentile and 97.5th quantiles. Panels A and B are for MAR data, when indicators are not included in the outcome simulation and when indicators are included in the outcome simulation. Panels C and D are for MNAR data, when indicators are not included in the outcome simulation and when indicators are included in the outcome simulation. AUC: area under the receiver operating characteristic curve; MAR: missing at random; MNAR: missing not at random.

We compare AUROC values for complete case analysis with the imputation methods (indicators included vs not included) in Figure 4A-D. AUROCs for the methods within a simulated scenario were generally similar and close to 0.75. The complete case analysis had the largest spread of AUROC values, whereas imputation with or with no missing indicators had similar spread of AUROC values. The amount of missing data (20% or 50%) and the missing data assumption (MAR and MNAR) did not substantially impact the AUROC values, which were similar across these groups. Comparing models using missing indicators with those with no missing indicators, we observed overlap in the AUROC intervals.

## Discussion

This study investigated the performance of the missing indicator method in terms of imputation quality and model performance for longitudinal data under MAR and MNAR mechanisms and different amounts of missing data. The imputation quality was worse under MNAR, as the PFC was about 15% higher under MNAR and the NRMSE for continuous values were higher under MNAR. When data were MAR and MNAR, the inclusion of missing indicators in the imputation and outcome models had a minimal effect on AUROC, regardless of whether the indicators were included as inputs when simulating the outcome. Therefore, the results from our simulation of longitudinal data mimicking data from the EHR suggest that the missing indicator method may not improve imputation quality or model performance, even when data are MNAR. However, it does not seem that including missing indicators harms imputation quality or model performance either.

In all scenarios, AUROC from complete case analysis was similar to AUROC from the other models, but the range of values was largest. While complete case analysis had similar AUROC values to imputation, we would not generally advocate for the use of complete case analysis. The increased variability associated with complete case analysis compared with imputation approaches can result in loss of power. In addition, while this study is focused on prediction and does not report model parameter estimates, complete case analysis may result in biased model coefficient estimates when data are MAR or MNAR. If model interpretation is of interest, complete case analysis will likely result in bias in settings such as EHR data, where missingness is often informative.

It was somewhat surprising that the imputations for the simulated binary variables were poor, as demonstrated by the high rates of PFC in Figure 2. We were not expecting such high errors in the imputed values. We hypothesize that some of the error may be attributed to rounding to force the imputed values to be binary, as many imputation methods provide a probability for binary variables which then must be handled in the analysis. Although the accuracy of the binary variable imputations was poor in our simulations, our main focus was on whether or not missing indicators may be beneficial for imputation and modeling. Future work may investigate the accuracy of imputation methods for multilevel data, especially when the predictors contain a mix of binary and continuous variables.

Previous studies on the missing indicator method have shown conflicting results. Van Ness et al [14] found that when missingness is informative, the missing indicator method increases predictive performance of linear models and neural networks with mean imputation and other imputation methods. The authors simulated data using an informativeness parameter, which differs from our study. The only situation where the method harmed predictive performance was in high-dimensional data, where the addition of uninformative indicators led to overfitting. Sperrin and Martin [39] found that the method improves causal effect estimation when missingness is informative when combined with multiple imputation. Sisk et al [11] investigated the use of the missing indicator method in addition to both regression and multiple imputation to deal with nonignorable missing data in prediction modeling. Similar to Van Ness et al [14], Sisk et al [11] showed that the missing indicator method corrected bias but requires the assumption that the missing mechanism remains constant throughout the clinical prediction model pipeline, which may not be plausible because of how the likelihood of collection differs across providers.

The results of our study contribute to the growing body of literature aiming to provide guidance regarding the missing indicator method. Our simulation based on EHR data of falls in older adults using a longitudinal, repeated-measures setup suggested that the missing indicator method may not be beneficial in terms of imputation quality or model performance, but it also did not seem to cause harm. None of the previously described papers used longitudinal data when investigating the missing indicator method with a focus on prediction modeling, which may be a reason why the results of this paper differ from findings of the other papers mentioned. There is clearly debate as to the potential benefit and harm of the missing indicator method, and this paper provides guidance for longitudinal, repeated-measures data.

Our study should be considered within the context of its strengths and limitations. We used a simulation framework, which has multiple advantages that allow for the evaluation of statistical methods. A major strength of this study is the ability to define and control the missing mechanism. In practice, investigators can make assumptions regarding why data are missing, but there is no statistical test to decide whether data are MAR or MNAR. In this study, because the truth regarding the missing mechanism for each variable is known, no assumptions are made. The effectiveness of the missing indicator method can be evaluated and compared between the 2 mechanisms. In addition, because the true value of all variables is known, the imputations themselves can be evaluated for quality.

Despite the many strengths of our study, there are some limitations. One limitation of this study was the quality of imputations for the binary variables. With 45%‐60% of values being incorrectly classified, the imputation performed only slightly better than random guessing. This may have impacted how beneficial the missing indicators were in modeling. A future study could investigate how to boost imputation performance in longitudinal data, perhaps using machine learning imputation methods. Another limitation of the study is that time-dependent covariates were not considered. Future work may investigate the missing indicator method in this setting. Other limitations are related to the nature of simulation studies. Assumptions about the relationships between variables must be made, and these relationships are often oversimplified. The results may be sensitive to the parameter values chosen for the study; however, we completed a rigorous study based on a real-world scenario of falls in older adults. Future studies could evaluate how the addition of more visits, missed visits, dropout, and other missing patterns common in EHR data impacts results. In addition, a future simulation study could use an informativeness missing parameter such as that imposed in Van Ness’ analysis for MNAR scenarios.

The results of this study suggest that the inclusion of missing indicators in longitudinal data modeling does not seem to be beneficial for overall performance or imputation accuracy, as neither metric improved. However, inclusion of missing indicators does not appear to cause harm in terms of performance or imputation accuracy, as neither metric worsened. Future research may address whether the inclusion of missing indicators is useful in prediction modeling with longitudinal data in different settings, such as high-dimensional data analysis.

## Supplementary material

10.2196/64354 Multimedia Appendix 1 Data simulation for one run of the simulation study.

## Abbreviations

AUROC: area under the receiver operating characteristic curve
EHR: electronic health record
MAR: missing at random
MCAR: missing completely at random
MNAR: missing not at random
NRMSE: normalized root-mean-square error
PFC: proportion of falsely classified imputations
TRIPOD: Transparent Reporting of a Multivariable Prediction Model for Individual Prognosis or Diagnosis
