# Supplementary material for: Imputation and Missing Indicators for Handling Missing Longitudinal Data: Data Simulation Analysis Based on Electronic Health Record Data
Source: JMIR Med Inform. 2025 Mar 13;13:e64354. doi: 10.2196/64354 (PMC11924964; doi:10.2196/64354)
Supplement: Multimedia Appendix 1 [file medinform-v13-e64354-s001.docx]

Supplemental document - Variable Summaries

2024-06-27

## Data Generation

### Patient Level Binary Variables

The binary patient-level variables birth sex, hypertension, diabetes, dementia, hypertension, and urinary incontinence were first simulated.

Below is a table of tetrachoric correlation followed by a table of counts for the 250 patients.

| **Variable** | **Birth sex** | **Urinary incontinence** | **Hypertension** | **Dementia** | **Diabetes** |
| --- | --- | --- | --- | --- | --- |
| Birth sex | 1.000 | 0.357 | 0.155 | 0.397 | -0.159 |
| Urinary incontinence | 0.357 | 1.000 | 0.042 | 0.376 | 0.371 |
| Hypertension | 0.155 | 0.042 | 1.000 | 0.422 | 0.472 |
| Dementia | 0.397 | 0.376 | 0.422 | 1.000 | 0.496 |
| Diabetes | -0.159 | 0.371 | 0.472 | 0.496 | 1.000 |

Patient-level Variable Summary

|  | Overall (N=250) |
| --- | --- |
| **Diabetes** |  |
| No | 199 (79.6%) |
| Yes | 51 (20.4%) |
| **Dementia** |  |
| No | 209 (83.6%) |
| Yes | 41 (16.4%) |
| **Hypertension** |  |
| No | 92 (36.8%) |
| Yes | 158 (63.2%) |
| **Urinary Incontinence** |  |
| No | 215 (86.0%) |
| Yes | 35 (14.0%) |
| **Birth Sex** |  |
| Male | 120 (48.0%) |
| Female | 130 (52.0%) |

### Age and Visit-level continuous variables

The patient-level variable age was the simulated, followed by the visit-level variables BMI, gait speed, and single leg balance. In the below table, the visit-level variables are averaged for each patient.

Continuous Variable Summary

|  | Overall (N=250) |
| --- | --- |
| **Age** |  |
| Mean (SD) | 79.0 (4.66) |
| Median [Min, Max] | 79.0 [65.0, 90.0] |
| **BMI** |  |
| Mean (SD) | 28.2 (2.08) |
| Median [Min, Max] | 28.1 [22.8, 34.6] |
| **Gait speed** |  |
| Mean (SD) | 0.837 (0.211) |
| Median [Min, Max] | 0.850 [0.204, 1.35] |
| **Single leg balance** |  |
| Mean (SD) | 16.0 (4.14) |
| Median [Min, Max] | 16.3 [4.85, 30.4] |

###

### Medication variables

The visit-level variables pain medication and depression medication were then simulated.

Medication Summary

|  | Overall (N=250) |
| --- | --- |
| **Average number of years on pain medication** |  |
| Mean (SD) | 2.37 (1.30) |
| Median [Min, Max] | 2.00 [0, 5.00] |
| **Average number of years on depression medication** |  |
| Mean (SD) | 2.40 (1.35) |
| Median [Min, Max] | 2.00 [0, 5.00] |

### Noise variables

Noise, or junk, variables were simulated. Their distributions were chosen at random.

Junk Variable Summary

|  | Overall (N=250) |
| --- | --- |
| **Junk Variable 1** |  |
| Mean (SD) | 8.01 (1.77) |
| Median [Min, Max] | 8.10 [4.06, 13.3] |
| **Junk Variable 2** |  |
| Mean (SD) | 29.8 (7.04) |
| Median [Min, Max] | 29.7 [9.77, 48.0] |
| **Junk Variable 3** |  |
| Mean (SD) | 100 (4.76) |
| Median [Min, Max] | 100 [84.9, 111] |
| **Junk Variable 4** |  |
| Mean (SD) | 0.0612 (0.422) |
| Median [Min, Max] | 0.0909 [-1.14, 1.09] |
| **Junk Variable 5** |  |
| Mean (SD) | 79.1 (21.9) |
| Median [Min, Max] | 77.9 [15.5, 138] |

### Outcome simulation

Finally, the outcome variable was simulated.


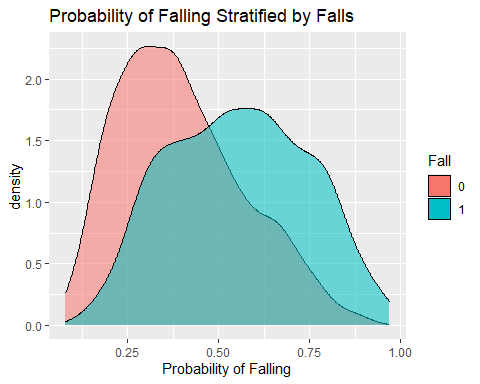


|  | Overall (N=1250) |
| --- | --- |
| **Falls** |  |
| No Fall | 649 (51.9%) |
| Fall | 601 (48.1%) |

Patient Characteristics Stratified by Falls

|  | No Fall (N=649) | Fall (N=601) | Overall (N=1250) |
| --- | --- | --- | --- |
| **Diabetes** |  |  |  |
| No.Diabetes | 567 (87.4%) | 428 (71.2%) | 995 (79.6%) |
| Diabetes.1 | 82 (12.6%) | 173 (28.8%) | 255 (20.4%) |
| **Dementia** |  |  |  |
| No.Dementia | 577 (88.9%) | 468 (77.9%) | 1045 (83.6%) |
| Dementia.1 | 72 (11.1%) | 133 (22.1%) | 205 (16.4%) |
| **Hypertension** |  |  |  |
| No.Hypertension | 289 (44.5%) | 171 (28.5%) | 460 (36.8%) |
| Hypertension.1 | 360 (55.5%) | 430 (71.5%) | 790 (63.2%) |
| **Urinary.Incontinence** |  |  |  |
| No.Urinary.Incontinence | 573 (88.3%) | 502 (83.5%) | 1075 (86.0%) |
| Urinary.Incontinence.1 | 76 (11.7%) | 99 (16.5%) | 175 (14.0%) |
| **Pain Medication** |  |  |  |
| No | 373 (57.5%) | 285 (47.4%) | 658 (52.6%) |
| Yes | 276 (42.5%) | 316 (52.6%) | 592 (47.4%) |
| **Depression Medication** |  |  |  |
| No | 348 (53.6%) | 303 (50.4%) | 651 (52.1%) |
| Yes | 301 (46.4%) | 298 (49.6%) | 599 (47.9%) |
| **Age** |  |  |  |
| Mean (SD) | 78.2 (4.61) | 79.9 (4.54) | 79.0 (4.65) |
| Median [Min, Max] | 78.0 [65.0, 90.0] | 80.0 [65.0, 90.0] | 79.0 [65.0, 90.0] |
| **BMI** |  |  |  |
| Mean (SD) | 28.0 (2.17) | 28.4 (2.31) | 28.2 (2.25) |
| Median [Min, Max] | 28.0 [21.9, 36.0] | 28.3 [22.3, 35.1] | 28.2 [21.9, 36.0] |
| **GS** |  |  |  |
| Mean (SD) | 0.862 (0.219) | 0.810 (0.229) | 0.837 (0.226) |
| Median [Min, Max] | 0.870 [0.250, 1.43] | 0.810 [0.0800, 1.45] | 0.850 [0.0800, 1.45] |
| **SLB** |  |  |  |
| Mean (SD) | 16.4 (4.42) | 15.5 (4.55) | 16.0 (4.50) |
| Median [Min, Max] | 16.6 [2.54, 33.8] | 15.6 [3.52, 29.5] | 16.1 [2.54, 33.8] |

## Modeling

A model summary is listed below.

|  | **Modeling Falls** | | |
| --- | --- | --- | --- |
| *Predictors* | *Odds Ratios* | *CI* | *p* |
| Intercept | 0.43 | 0.31 – 0.60 | **<0.001** |
| Age | 1.16 | 0.98 – 1.37 | 0.078 |
| BMI | 1.14 | 0.99 – 1.32 | 0.074 |
| Gait speed | 1.03 | 0.88 – 1.21 | 0.722 |
| Single leg balance | 0.82 | 0.72 – 0.94 | **0.005** |
| Birth sex | 1.36 | 0.99 – 1.87 | 0.059 |
| Diabetes | 1.96 | 1.28 – 3.00 | **0.002** |
| Dementia | 1.52 | 1.00 – 2.32 | 0.051 |
| Hypertension | 1.48 | 1.08 – 2.04 | **0.016** |
| Urinary incontience | 1.04 | 0.68 – 1.60 | 0.857 |
| Pain medication | 1.37 | 1.07 – 1.77 | **0.014** |
| Depression medication | 0.98 | 0.76 – 1.26 | 0.874 |
| Junk 1 | 1.00 | 0.88 – 1.13 | 0.974 |
| Junk 2 | 0.97 | 0.85 – 1.09 | 0.581 |
| Junk 3 | 1.06 | 0.94 – 1.20 | 0.327 |
| Junk 4 | 0.96 | 0.84 – 1.08 | 0.470 |
| Junk 5 | 1.10 | 0.97 – 1.24 | 0.150 |
| **Random Effects** | | | |
| σ^2^ | 3.29 | | |
| τ_00_ _patient_id_ | 0.29 | | |
| ICC | 0.08 | | |
| N _patient_id_ | 250 | | |
| Observations | 1250 | | |
| Marginal R^2^ / Conditional R^2^ | 0.114 / 0.186 | | |
